# PSRNet: a phase-guided frequency-domain structure reconstruction network for RGB-T salient object detection

**DOI:** 10.3389/fnbot.2026.1863193

**Published:** 2026-07-06

**Authors:** Feng Xie, Junhong Zhou, Feng Gao, Xinqiang Ma, Chunyu Yang

**Affiliations:** 1 School of Mathematics and Artificial Intelligence, Chongqing University of Arts and Sciences, Chongqing, China; 2 School of Foreign Languages, Jilin Agricultural Science and Technology University, Jilin, China

**Keywords:** amplitude-weighted structural alignment, boundary recovery, bounded structure reconstruction, frequency-domain learning, latent phase consistency, multimodal perception, RGB-T salient object detection

## Abstract

**Introduction:**

RGB-T salient object detection remains challenging because visible and thermal features often show structural shifts, weak thermal boundaries, and background interference.

**Methods:**

This study proposes PSRNet, a phase-guided frequency-domain structure reconstruction network. RGB and thermal features are decomposed into amplitude and latent-phase components in the Fourier domain. Reliable cross-modal structural cues are aligned through amplitude-weighted phase consistency, and boundary-related high-frequency responses are reconstructed with a bounded Gaussian high-pass gate before adaptive phase-modulated fusion and multi-scale supervision.

**Results:**

On VT1000, PSRNet achieved an S-measure of 0.903, an MAE of 0.028, and an F-measure of 0.794 at threshold 0.80, with a boundary IoU of 0.862.

**Discussion:**

The results indicate improved structural preservation and boundary recovery under challenging RGB-T conditions.

## 1 Introduction

Salient object detection (SOD) aims to identify visually distinctive objects and provide compact foreground representations for downstream tasks such as autonomous driving, intelligent surveillance, human-computer interaction, and target localization (Wang et al., 2024; Song et al., 2024). Conventional RGB-based SOD methods benefit from rich texture and color cues, but their reliability often decreases under low illumination, occlusion, and cluttered backgrounds (Rasheed et al., 2025; Fu et al., 2025). Thermal infrared imaging offers complementary radiation-based cues and is less sensitive to visible-light degradation, making RGB-T SOD an important direction for robust multimodal visual perception (Tian et al., 2025; Li et al., 2025). Recent RGB-T studies have improved cross-modal interaction and feature aggregation, yet stable structural representation and precise boundary recovery remain difficult in complex scenes (Wang et al., 2023; Luo et al., 2024; Chen et al., 2024; Peng et al., 2025).

The difficulty arises from both modality heterogeneity and structural degradation. RGB features may lose discriminative texture in poorly illuminated regions, whereas thermal features often contain coarse object contours and blurred edges (Hu et al., 2023; Zou and Yang, 2023). The two modalities also differ in imaging mechanism, spatial response, and feature distribution, which can introduce cross-modal misalignment during fusion (Yuan et al., 2024; Jin et al., 2024). When redundant background responses are fused without explicit structural constraints, salient boundaries become less separable and target morphology may be incomplete (Zhao et al., 2024; Chen et al., 2023). Therefore, RGB-T SOD requires not only semantic fusion but also a structure-aware mechanism capable of preserving boundary topology across modalities.

Existing RGB-T SOD methods can be roughly grouped into convolutional fusion, attention-based fusion, transformer-based interaction, edge-guided refinement, and relational modeling. Convolutional fusion methods concatenate or aggregate bimodal features but may underrepresent modality-specific structural differences (Xu et al., 2024; Deng et al., 2024). Attention and transformer models strengthen long-range dependencies and adaptive weighting, although fine boundary details may still be weakened after repeated downsampling (Cong et al., 2022; Qiu et al., 2024). Edge-guided and frequency-enhanced approaches explicitly emphasize contours, but they often depend on edge maps that are unstable in thermal images or noisy backgrounds (Wu et al., 2024; Li et al., 2023). These limitations suggest that cross-modal structure should be modeled at a more intrinsic representational level rather than only through spatial-domain aggregation.

Motivated by the classical observation that Fourier phase is closely related to spatial arrangement and contour information, this paper proposes PSRNet, a phase-guided frequency-domain structure reconstruction network for RGB-T SOD. We do not assume that the raw-image phase is preserved unchanged after nonlinear deep transformations. Instead, phase is treated as a latent structural descriptor extracted from resolution-preserving intermediate features and used as an auxiliary cue for contour alignment. PSRNet decomposes RGB and thermal features into amplitude and phase components, aligns reliable cross-modal structural cues through an amplitude-weighted phase consistency constraint, and reconstructs boundary-sensitive high-frequency responses through a bounded Gaussian high-pass gate. The reconstructed structure is injected back into the spatial stream through adaptive phase-modulated fusion, improving foreground integrity while reducing background interference. This formulation is aligned with the journal scope on robust multimodal perception, interpretable deep representation, and reliable image analysis under challenging sensing conditions.

The main contributions of this study are summarized as follows:

1. A frequency-domain representation branch is introduced into RGB-T SOD to explicitly separate energy-related amplitude information from structure-related phase information in bimodal deep features.
2. An amplitude-weighted cross-modal phase consistency constraint is formulated to align RGB and thermal structural cues while reducing the influence of low-amplitude phase singularities and noisy background regions.
3. A bounded Gaussian high-pass-guided structure reconstruction operator and adaptive phase-modulated fusion unit are designed to recover boundary-related high-frequency details without introducing unconstrained phase displacement in the spatial domain.
4. Comprehensive experiments, robustness analysis, and reorganized ablation evidence are provided to evaluate structural preservation, cross-modal alignment, boundary recovery, and degradation resistance under low illumination, occlusion, and noise.

## 2 Methods

### 2.1 PSRNet overall framework design

The RGB-T SOD task requires joint use of visible-light texture cues and thermal radiation cues. Because the two modalities have different sensing mechanisms and response statistics, directly fusing their spatial-domain features may produce structural drift, boundary ambiguity, and redundant background activation. PSRNet therefore embeds a frequency-domain structure modeling branch into the dual-encoder and decoder pipeline. The branch does not replace spatial feature learning; rather, it provides an explicit structural regularization path in which phase-related information is aligned, reconstructed, and returned to the spatial feature stream for saliency prediction.

The network input consists of RGB images $I^{\mathrm{rgb}}$ and thermal infrared images $I^{t}$ , both maintaining the same spatial resolution. These images are then fed into separate modality coding branches for hierarchical feature extraction. The encoder constructs progressively abstract feature representations through multi-layer convolutional structures, preserving edge and texture details in lower-level features and forming semantic expressions in higher-level features. Let the encoding network be a function E(·) , and the deep feature representations of RGB and thermal infrared be denoted as follows:


$$F^{\mathrm{rgb}}=ℰ(I^{\mathrm{rgb}}),F^{t}=ℰ(I^{t})$$ (1)

In Equation 1, $F^{\mathrm{rgb}}$ and $F^{t}$ denote the deep feature tensors output by the RGB and thermal branches, respectively; ε(·) denotes the feature-encoding mapping with shared structure and independent parameters, $I^{\mathrm{rgb}}$ and $I^{t}$ denote the input RGB and thermal infrared images, respectively.

While spatial domain features retain semantic expressiveness, they lack explicit characterization of structural essence. Therefore, PSRNet introduces a frequency domain modeling path in deep feature layers, mapping bimodal features to the frequency space to resolve structural information. Feature tensors are mapped to a frequency domain representation through a two-dimensional discrete Fourier transform, the computation of which is expressed as follows:


$$ℱ(u,v)=\sum_{x=0}^{H-1}\sum_{y=0}^{W-1}F(x,y)\;e^{-j2\pi (\frac{\mathrm{ux}}{H}+\frac{\mathrm{vy}}{W})}$$ (2)

In Equation 2, F(x,y) denotes the spatial-domain feature response at location (x,y) ; (u,v) denotes the frequency-domain coordinate; H and W denote the feature-map height and width, respectively; and j is the imaginary unit. The frequency domain feature consists of two parts: amplitude and phase. Amplitude characterizes the energy distribution, and phase describes the structural positional relationships. Therefore, the complex spectrum is further decoupled as follows:


A(u,v)=∣ℱ(u,v)∣,P(u,v)=arg(ℱ(u,v)) (3)

In Equation 3, A(u,v) denotes the amplitude spectrum, which reflects the energy intensity of frequency components, whereas P(u,v) denotes the phase spectrum, which captures structural boundary and spatial-location relationships. Phase information plays a core role in structural representation; therefore, PSRNet establishes a structural modeling path around the phase components.

Based on frequency domain decomposition, the network constructs a phase structure reconstruction module. This module aligns structural information through cross-modal phase consistency constraints and recovers high-frequency structural components in the frequency domain. After structural modulation of the phase features, the module reassembles them into a complex spectrum, which is then mapped back to the spatial domain via inverse Fourier transform. The process is represented as follows:


$$\hat{F}(x,y)=F^{-1}(A(u,v)\cdot e^{j\hat{P}(u,v)})$$ (4)

In Equation 4, $\hat{F}(x,y)$ denotes the spatial-domain feature after structural reconstruction, $F^{-1}(.)$ denotes the inverse Fourier transform, $\hat{P}(u,v)$ denotes the adjusted phase component, and A(u,v) denotes the original amplitude spectrum. This process allows frequency domain structural information to flow back into the spatial feature representation, thereby enhancing the structural and boundary information of salient regions.

The reconstructed spatial features and the original spatial features are jointly represented in a fusion unit. A phase modulation mechanism is used to adjust the spatial response, enhancing the structural response of the target region while suppressing background noise. The fused features are then decoded step-by-step to restore the spatial resolution, ultimately generating a salient target prediction map S . Let the decoding function be D(·) , and the final prediction result be expressed as:


$$S=D({\hat{F}}^{\mathrm{rgb}},{\hat{F}}^{t})$$ (5)

In Equation 5, S denotes the predicted saliency map; ${\hat{F}}^{\mathrm{rgb}}$ and ${\hat{F}}^{t}$ denote the reconstructed RGB and thermal feature representations, respectively; and D(·) denotes the multilayer decoder.

Overall, PSRNet follows an information flow of spatial feature encoding, frequency-domain amplitude-phase decomposition, cross-modal phase alignment, phase-guided structure reconstruction, spatial-domain feedback fusion, and saliency map prediction. This design keeps the semantic representation capacity of the bimodal encoders while explicitly strengthening boundary-sensitive structural cues that are otherwise weakened during multi-layer propagation.

**Figure 1:**
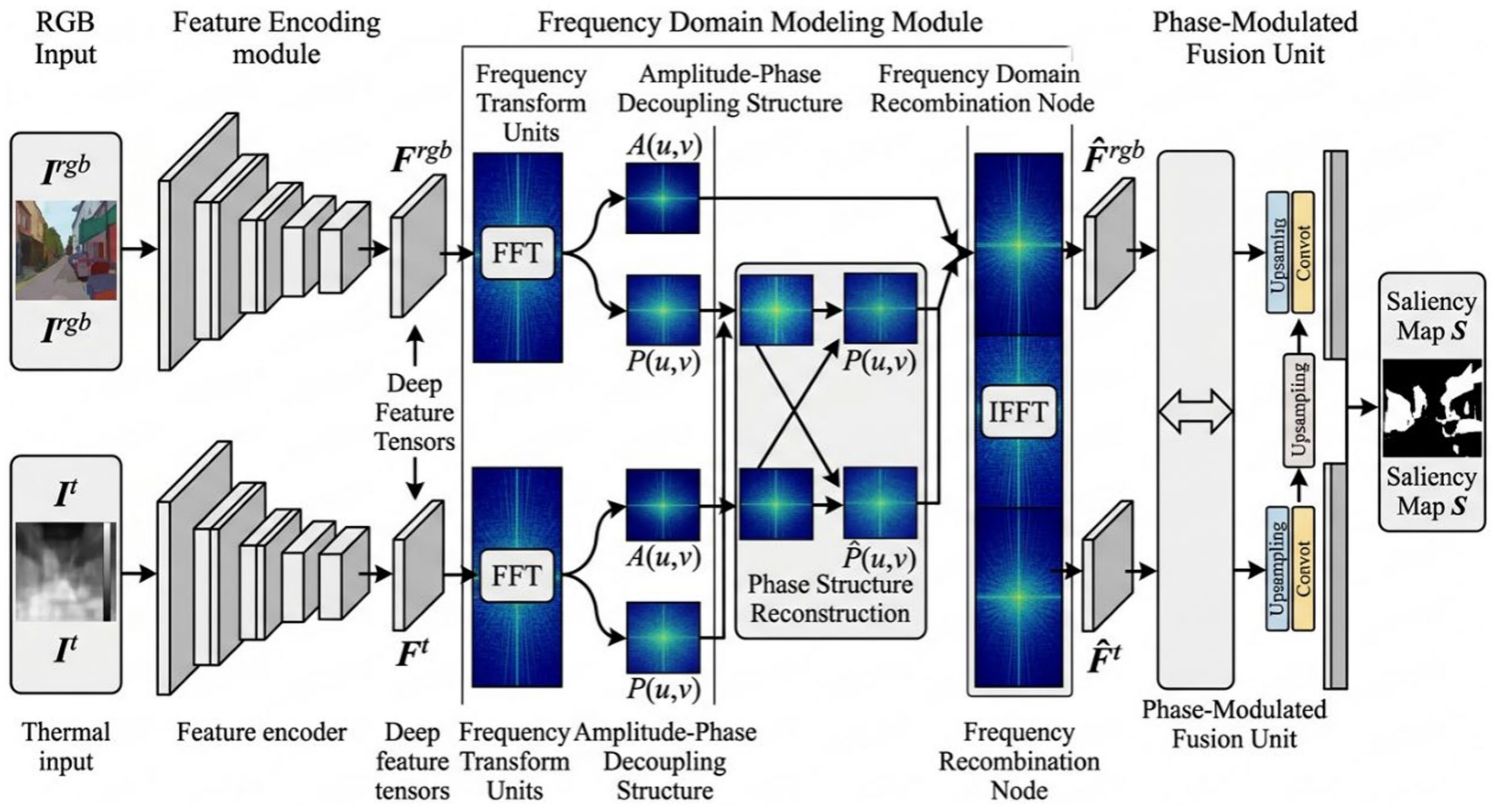
PSRNet overall framework diagram.

Figure 1 summarizes the PSRNet architecture. RGB and thermal inputs are encoded through parallel branches, and their deep features are transformed into frequency-domain representations. Amplitude and phase components are then decoupled; the phase branch is used for cross-modal structural alignment and reconstruction, while the amplitude branch preserves energy distribution. The reconstructed structural cues are mapped back to the spatial domain and fused with the original features before progressive decoding. Detailed module definitions are provided in Sections 2.2–2.5.

### 2.2 Frequency domain decoupling and phase representation modeling

To characterize the structural information in deep features, the network performs frequency domain mapping on the bimodal spatial domain features during the encoding stage, transforming the feature representation output by the convolutional encoder to the complex spectral space. Let $F^{r}\in R^{C\times H\times W}$ and $F^{t}\in R^{C\times H\times W}$ denote the deep features from the RGB and thermal infrared branches, respectively. Their frequency-domain representations are constructed using a two-dimensional DFT, yielding complex spectra ${ℱ}^{r}$ and ${ℱ}^{t}$ , whose mapping relationship is defined as.


$$ℱ(u,v)=\sum_{x=0}^{H-1}\sum_{y=0}^{W-1}F(x,y)\;e^{-j2\pi (\frac{\mathrm{ux}}{H}+\frac{\mathrm{vy}}{W})},$$ (6)

As shown in Equation 6, where F(x,y) represents the response of the spatial domain input feature at the location (x,y) , (u,v) represents the frequency domain coordinates, H and W represents the height and width of the feature map, respectively, j and is an imaginary unit. This transformation maps the local convolution response in the spatial domain to the global frequency distribution in the frequency domain, so that the structural contours and texture boundaries exhibit clear high-frequency responses in the spectrum.

After obtaining the complex spectrum, amplitude and phase decoupling operations are performed on the frequency domain features to independently model the structural information and energy distribution. For any frequency domain coefficient ℱ(u,v)∈C , its amplitude and phase components are defined as follows:


A(u,v)=∣F(u,v)∣,Φ(u,v)=atan2Im(F(u,v))Re(F(u,v)), (7)

As shown in Equation 7, where A(u,v) represents the amplitude value of the frequency component, Φ(u,v) represents the phase angle, Re(·) and Im(·) represent the real and imaginary parts of the complex number, respectively. The amplitude component reflects the intensity distribution of different frequency components, while the phase component determines the spatial arrangement relationship between the frequencies and plays a dominant role in maintaining the image structure contour. Through this decoupling operation, the boundary information and shape contour in the bimodal features are concentrated and encoded into the phase component, providing a stable representation basis for subsequent structure alignment and reconstruction.

To enhance the structural representation capability of the phase components, the decoupled phase features are normalized and mapped to construct a phase structure representation. Let the phase component be Φ(u,v) , and its normalized form be defined as.


$$\hat{\Phi }(u,v)=\frac{\Phi (u,v)-\mu_{\Phi }}{\sigma_{\Phi }+\epsilon },$$ (8)

As shown in Equation 8, where $\mu_{\Phi }$ and $\sigma_{\Phi }$ denote the mean and standard deviation of the phase distribution, respectively, and ϵ is a stabilizing term used to avoid division by zero. The normalized phase features form a unified scale across different modes, making the high-frequency structural region exhibit a more concentrated response distribution in the spectrum and reducing the interference of amplitude differences on structural modeling. This process explicitly separates structural and energy information in the frequency domain, making phase features the core carrier for cross-modal structural expression.

Through the frequency-domain mapping and decoupling process, RGB and thermal features form amplitude and phase representations in a shared spectral space. In this work, the phase spectrum is not regarded as the raw-pixel phase preserved through nonlinear activation. It is instead used as a latent structural descriptor that captures relative spatial arrangement in intermediate feature maps. To reduce distortion caused by nonlinear activations, pooling, and downsampling, the frequency branch is applied to resolution-preserving intermediate features and is jointly supervised by boundary-sensitive and phase-consistency losses. This design allows the latent phase to provide structural guidance for contour alignment while the spatial-domain branch retains semantic discrimination.

**Figure 2:**
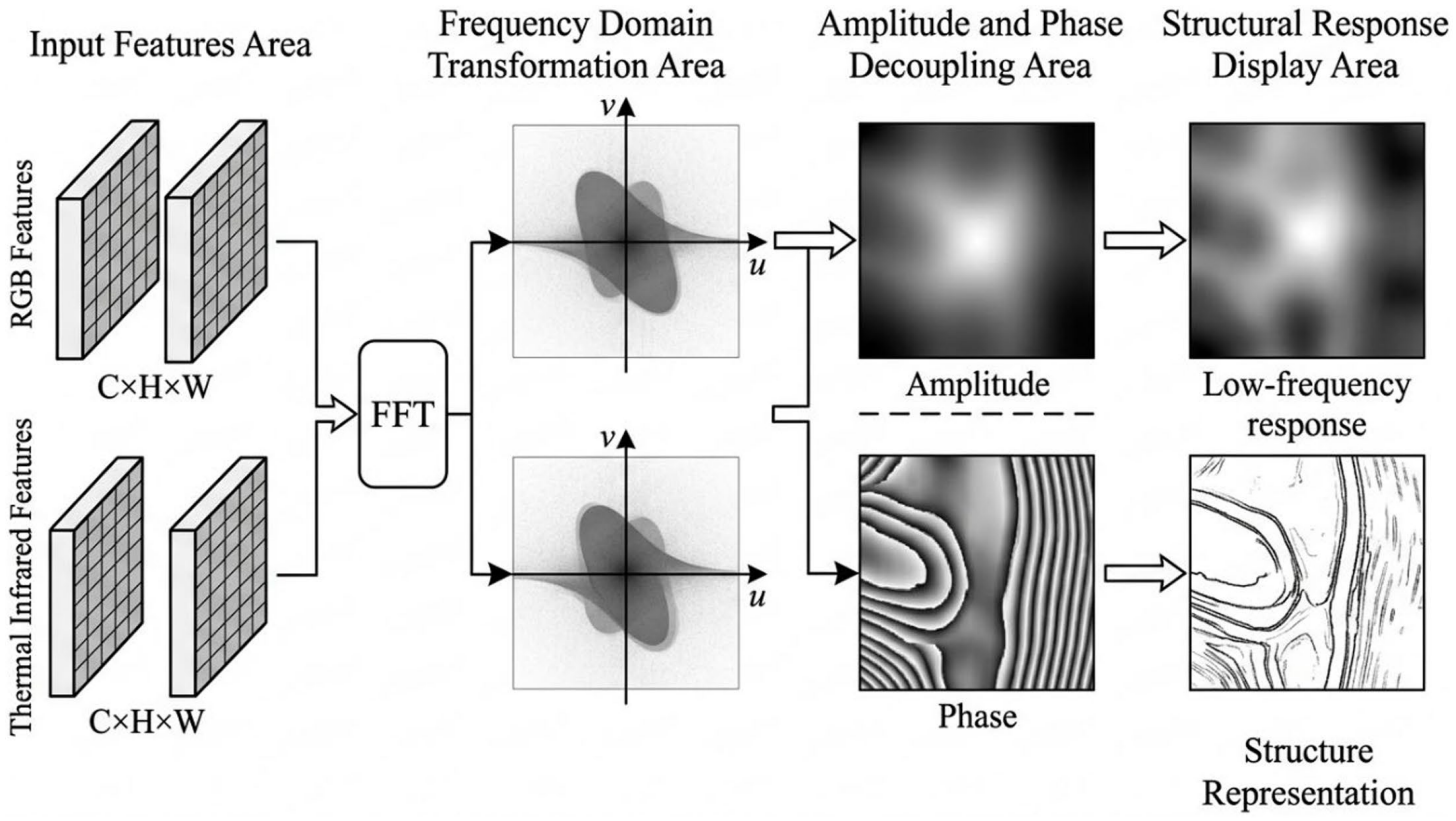
Schematic diagram of frequency domain decomposition and phase response.

**Figure 3:**
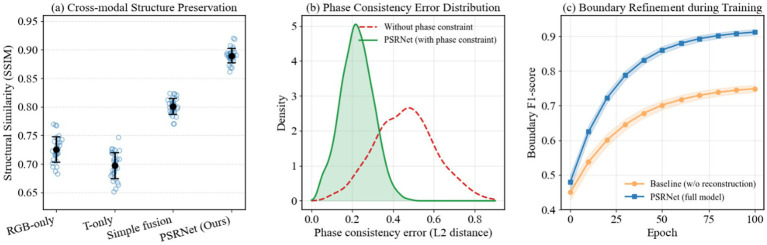
Analysis of cross-modal fusion effect. **(a)** The structures of different fusion strategies are kept in contrast. **(b)** Phase consistency error distribution. **(c)** Boundary F1 curve during training.

**Figure 4:**
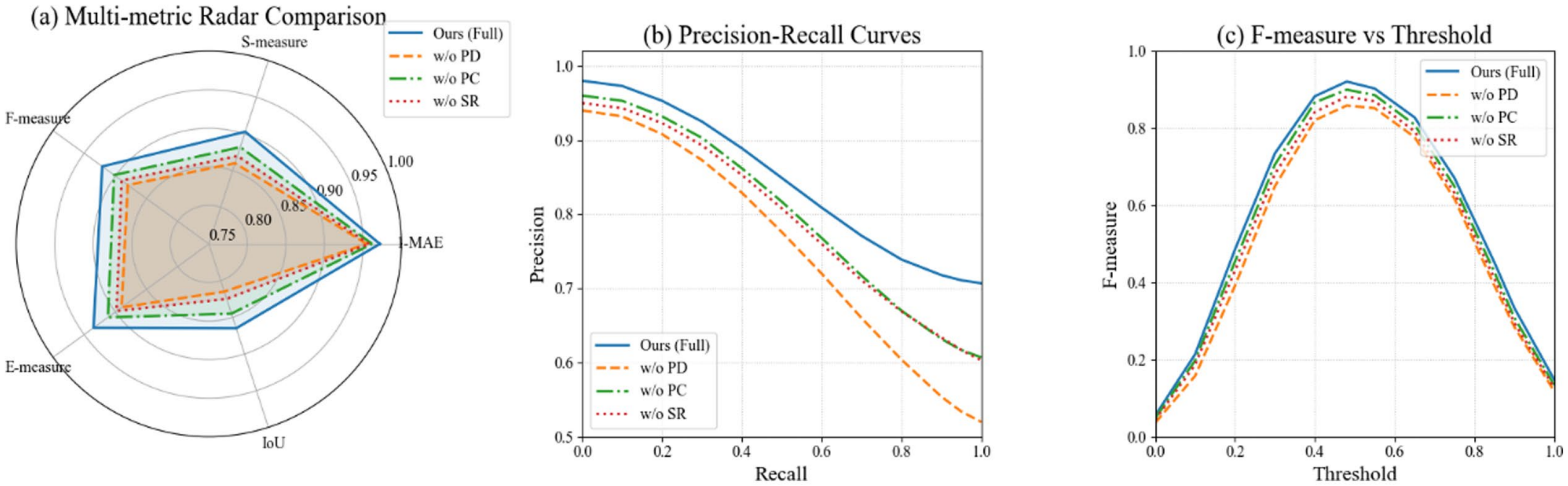
Module validity and ablation analysis. **(a)** Multi-metric ablation radar comparison. **(b)** Precision-Recall curve. **(c)** F-measure curve as a function of threshold.

Additional empirical support is provided through the frequency-response visualization in Figure 2, the phase-consistency error distribution in Figure 3b, and the ablation evidence in Figure 4. These analyses show that the phase-guided branch is interpreted as structure guidance rather than exact inverse recovery of raw images. Low-amplitude regions are down-weighted in the phase loss to avoid phase singularities, and the reconstruction effect is evaluated by boundary recovery and structural preservation rather than by pixel-level spectral reconstruction alone.

Figure 2 illustrates the frequency domain decoupling and phase representation modeling process in a horizontal flow diagram. On the left are the deep spatial domain features of RGB and thermal infrared, represented by parallel rectangular feature blocks with labeled dimensions to demonstrate that the dual-modal input maintains an independent structure before entering the frequency domain. The two feature paths are connected to the frequency domain transformation unit via arrows. This region is represented by a two-dimensional spectrum matrix C×H×W with v frequency axes u , where the center represents the low-frequency region and the periphery represents the high-frequency region, reflecting the mapping relationship between the spatial response and the global frequency distribution. The frequency domain features are decomposed into two parallel paths in the middle. The upper path corresponds to the amplitude component, using a smooth grayscale distribution to represent energy changes, while the lower path corresponds to the phase component, using a structural texture with a clear contour to represent the spatial arrangement. The two paths are separated by forked arrows and dashed lines to demonstrate the decoupling structure of amplitude and phase. The right-hand area presents the decoupled structural response results. The amplitude component is connected to the low-frequency energy distribution map, showing an overall smooth change, while the phase component is connected to the structural contour response map, showing clear boundary and detail information. The two are arranged side by side to highlight the dominant role of the phase component in the structural expression, fully demonstrating the process of mapping bimodal features from the spatial domain to the frequency domain and enhancing structural information through phase modeling.

### 2.3 Cross-modal phase consistency constraint mechanism

Significant differences exist between RGB and thermal infrared modes in their imaging mechanisms and radiometric responses, leading to a shift in the spatial structure representation of bi-branch features. The phase component in the frequency domain representation directly determines the spatial structure and boundary positions. Therefore, a cross-modal phase consistency constraint is established at the frequency domain level to align the structural information of the two modes and suppress structural drift introduced by modal differences. By decoupling the deep features of RGB and thermal infrared in the frequency domain, their corresponding phase representations are obtained. Subsequently, a cross-modal phase alignment path is constructed, enabling the two modes to form a consistent frequency domain response in their structural representations.

Let the deep features from the RGB branch and the thermal infrared branch be $F_{r}\in R^{C\times H\times W}$ and, respectively $F_{t}\in R^{C\times H\times W}$ , where C represents the number of channels, H and W represent the spatial dimensions of the feature maps, respectively. Applying a two-dimensional Fourier transform to the bimodal features, we can map them to the frequency domain as follows:


$${ℱ}_{r}=ℱ(F_{r}),{ℱ}_{t}=ℱ(F_{t})$$ (9)

As shown in Equation 9, where ℱ(·) represents the two-dimensional discrete Fourier transform operator, ${ℱ}_{r}$ and ${ℱ}_{t}$ are the complex representations of the RGB and thermal infrared modes in the frequency domain, respectively. Amplitude and phase decomposition is performed on the complex features in the frequency domain to obtain:


$${ℱ}_{r}=A_{r}\cdot e^{j\phi_{r}},{ℱ}_{t}=A_{t}\cdot e^{j\phi_{t}}$$ (10)

As shown in Equation 10, where $A_{r}$ and $A_{t}$ represent the bimodal amplitude components, $\phi_{r}$ and $\phi_{t}$ represent the corresponding phase components, j with being the imaginary unit. The phase components $\phi_{r},\phi_{t}\in R^{C\times H\times W}$ describe the geometric distribution information of the spatial structure and have more stable structural consistency characteristics between different modes.

To reduce cross-modal structural discrepancy, PSRNet introduces an amplitude-weighted phase consistency constraint. Because phase values in low-amplitude regions are sensitive to noise and may become numerically unstable, the revised formulation weights the phase-alignment gradient by the corresponding spectral magnitude. This prevents background-dominated or weak-response frequencies from contributing the same optimization strength as boundary-related frequencies.


$$L_{\mathrm{pc}}^{\left(l\right)}=\frac{1}{M_{l}}\sum_{c,u,v}w_{l}(u,v)|\sin(\phi_{l}^{R}(c,u,v)-\phi_{l}^{T}(c,u,v))|$$ (11)

As shown in Equation 11, L_pc denotes the cross-modal phase consistency loss. *ϕR* and *ϕT* denote RGB and thermal phase values at scale l, channel c, and frequency coordinate (u, v), while Wl(u, v) is the normalized amplitude-derived reliability weight. The sine-based circular distance is retained for periodicity, and the equivalent complex-domain form |exp.(j $\phi_{l}^{R}$ )-exp(j $\phi_{l}^{T}$ )|2 is discussed as a stable implementation alternative. This harmonizes the phase-loss definitions used in Equations 11 and 19.

During phase alignment, a cross-modal interaction operator constructs a shared structural phase representation through adaptive reliability weighting rather than a fixed global coefficient. A lightweight attention branch estimates input-dependent weights αR and αT from RGB and thermal feature statistics, followed by softmax normalization. This allows the model to reduce the contribution of unreliable modalities under low-light, thermal crossover, occlusion, or noise.


$$\phi^{s}=\alpha_{R}\phi^{R}+\alpha_{T}{\phi^{T}}_{,}\;\;\alpha_{R}+\alpha_{T}=1$$ (12)

As shown in Equation 12, the shared phase *ϕ*S is obtained by *αR*. *ϕR* + *αT*· *ϕT*, where α*r* and α*t* vary across samples and feature locations. The notation has been revised to avoid reusing alpha for later spatial modulation; beta is reserved for the phase-modulated fusion unit in Equation 16.

Through amplitude-weighted phase consistency and adaptive phase interaction, RGB and thermal features achieve more stable structural alignment in the frequency domain. Modal differences in background regions are suppressed, while target contours and internal structures are reinforced. In the FFT/IFFT implementation, reflection padding and a smooth window are used before spectral transformation, and the valid region is restored after inverse transformation. This practical treatment reduces spectral leakage, periodic-boundary discontinuity, and ringing artifacts without changing the saliency labels or evaluation protocol.

**Figure 5:**
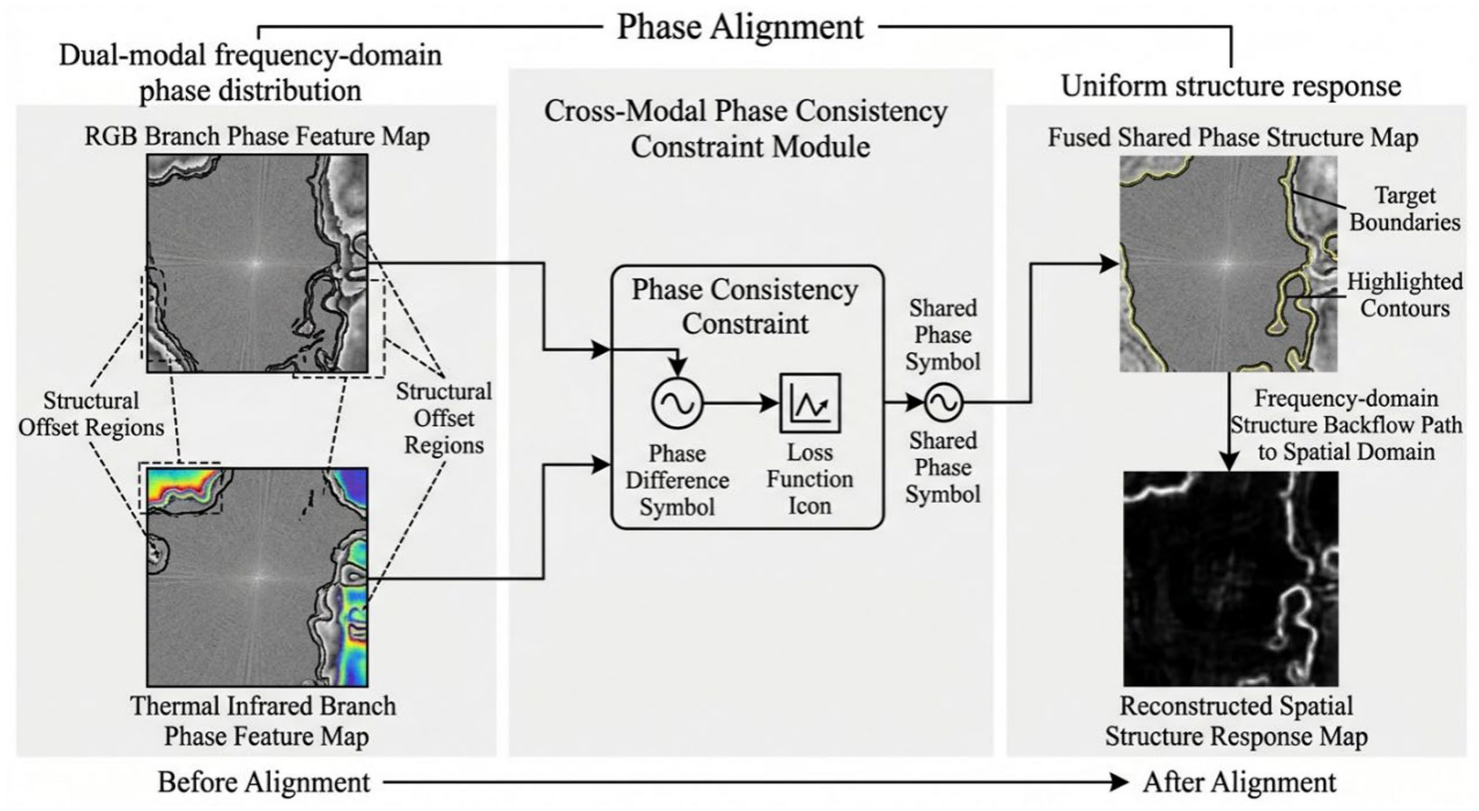
Schematic diagram of cross-modal phase alignment results.

Figure 5 illustrates the structural response changes before and after cross-modal phase consistency constraints. The overall layout uses a left–right comparison, consisting of three parts: before phase alignment, the phase consistency constraint module, and after phase alignment. The left area shows the original phase distributions of the RGB and thermal infrared modes. The two frequency domain phase maps, arranged vertically, correspond to the dual-modal structural responses. Different texture densities and contour lines indicate differences in target boundary positions, and dashed lines mark structural offset positions, reflecting inconsistencies in the frequency domain structural representation between modes. The middle area is the cross-modal phase consistency constraint module. Arrows from the dual-modal phase maps are simultaneously input to the phase alignment unit. The module internally uses phase difference calculation symbols and constraint identifiers to represent the consistency loss construction process. Simultaneously, the module outputs the fused shared phase representation, demonstrating the phase interaction and structural alignment path. The right-hand area displays the structural response results after phase alignment. The upper part shows the fused shared phase distribution, and the lower part shows the structural response diagram after the shared phase flows back to the spatial domain. The two are connected by a solid arrow, indicating the process of propagation of the frequency domain structure to the spatial domain. The aligned target boundary is presented with a continuous highlighted outline, which contrasts with the unaligned result on the left, reflecting the improvement in structural consistency and boundary integrity. The overall information flow is organized from left to right, clearly reflecting the optimization effect of cross-modal phase alignment on structural expression.

### 2.4 Phase-guided structural reconstruction and fusion mechanism

After amplitude-phase decoupling, PSRNet uses the phase representation as structural guidance and the amplitude representation as the carrier of spectral energy. To avoid arbitrary phase rotation that could cause spatial displacement, the reconstruction is formulated as a bounded structure-guided gate rather than an unconstrained additive phase shift. Thus, boundary-sensitive high-frequency components are enhanced within a controlled range, while the global spatial layout and salient-object topology are preserved.

Let the fused feature from the RGB and thermal infrared branches be represented as $F\in R^{C\times H\times W}$ , where C , H , W and represent the number of channels, height, and width, respectively. This feature has been mapped to the frequency domain and decomposed into decomposed into amplitude A and phase P components in the preceding stages. In the frequency domain, the high-frequency region carries the target boundary and fine-grained structural information, while the low-frequency region mainly describes the overall contour and brightness distribution. To enhance the structural representation, a high-frequency structure enhancement operator is introduced to guide the reconstruction of the phase information. The frequency domain structure reconstruction process is represented as.


$$F_{r}(u,v)=A(u,v)\cdot e^{i\;\tilde{P}(u,v)}$$ (13)

As shown in Equation 13, $F_{r}(u,v)$ represents the frequency domain characteristics after structural reconstruction, A(u,v) represents the original amplitude spectrum, $\tilde{P}(u,v)$ represents the phase component after structural enhancement, represents the $e^{i\tilde{P}(u,v)}$ complex exponential form of the spectrum u,v constructed from the phase, and represents the frequency domain coordinate position. This expression achieves the reconstruction of the frequency domain structure distribution by retaining the original amplitude information and adjusting the phase structure.

The structure-guided enhancement is controlled by a Gaussian high-pass response H(u, v). Let D(u, v) denote the normalized distance between frequency coordinate (u, v) and the spectral center. The response is defined as H(u, v) = 1 − exp.[−D(u, v)^2/(2 σ2)], where σc controls the cutoff bandwidth. This soft high-pass form avoids the discontinuities of an ideal high-pass filter and reduces ringing artifacts during inverse transformation. To make the boundedness explicit, the actual phase update is constrained by a saturating gate: ϕ(u, v) = ∆tanh(H(u, v) Gθ(A(u, v), φ(u, v))), so that |ϕ(u, v)| < = ∆. The hyperparameter delta_max is set to a small value in implementation, and the updated phase is wrapped to the principal interval before spectral recomposition.


$$\begin{array}{c} H(u,v)=1-\exp(-\frac{D{\left(u,v\right)}^{2}}{2\sigma_{c}^{2}}),\\ \Delta \phi (u,v)=\delta_{\max}\tanh[H(u,v)G_{\theta }(A(u,v),\phi (u,v))],\\ \tilde{\phi }(u,v)=\text{wrap}(\phi (u,v)+\Delta \phi (u,v)),|\Delta \phi (u,v)|\leq \delta_{\max} \end{array}$$ (14)

As shown in Equation 14, the reconstruction uses H(u,v) is the Gaussian high-pass response, D(u,v) is the normalized distance from frequency coordinate (u,v) to the spectral center, σc is the cutoff-bandwidth parameter, ∆_max is the maximum allowed phase perturbation, θ(·) is the learnable structure-guided modulation function, A(u,v) is the amplitude component, Φ(u,v) is the original phase component, Delta Φ(u,v) is the bounded phase update, and Φ(u,v) is the wrapped updated phase.

Implementation note. The bounded gate is applied only to the high-frequency structural response and not to the global low-frequency phase. Reflection padding and smooth-window preprocessing are used before FFT/IFFT, and the valid region is restored after inverse transformation. This avoids boundary discontinuities and keeps the reconstructed response localized around salient contours.

After the frequency domain structure is reconstructed, the reconstructed features are mapped back to the spatial domain using inverse Fourier transform to obtain the structure enhancement features $F_{s}$ , which are expressed as follows:


$$F_{s}(x,y)={ℱ}^{-1}(F_{r}(u,v))$$ (15)

As shown in Equation 15, $F_{s}(x,y)$ represents the structural enhancement feature in the spatial domain, ${ℱ}^{-1}(\cdot )$ represents the two-dimensional inverse Fourier transform operator, and (x,y) represents the spatial domain coordinate position. After this transformation, the enhanced phase structure in the frequency domain manifests as a clearer boundary and structural response in the spatial domain.

To effectively integrate the reconstructed structural information into the original feature representation, a phase-modulated fusion unit (PMFU) is designed to perform structural modulation on the spatial domain features. Let the original fused feature be F and the structurally enhanced feature be $F_{s},$ and the fusion process be represented as follows:


$$F_{f}=F+\beta \cdot (F⊙\sigma (F_{s}))$$ (16)

As shown in Equation 16, Here, F_f denotes the final fused feature, F denotes the original fused feature, F_s denotes the structurally enhanced feature, beta is the spatial structure modulation coefficient, odot denotes element-wise multiplication, and σ(·) denotes the normalized activation function. Please correct “Fand” to “F and” and use F_f consistently instead of F_out.

**Figure 6:**
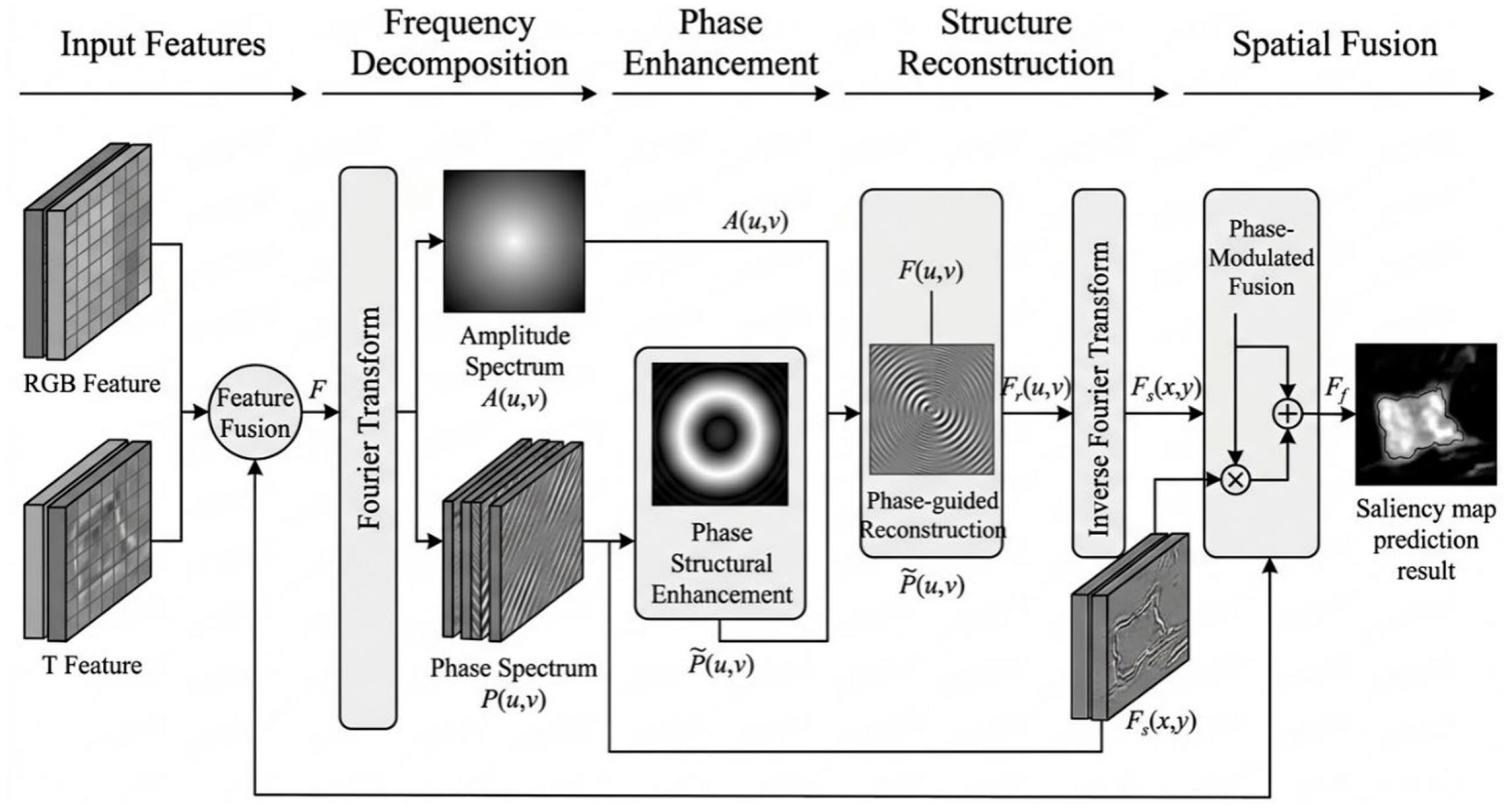
Schematic diagram of structural reconstruction and spatial response.

In the spatial domain, structural enhancement features generate high response values in the target boundary region, resulting in a clearer structural gradient in the feature map at the contour location. This structural gradient forms a stable boundary representation in the subsequent saliency map prediction stage, thereby improving the structural integrity problem under complex background conditions. The structural reconstruction module is embedded after dual-modal feature fusion, and achieves salient region enhancement and background interference suppression through a joint mechanism of frequency domain structure recovery and spatial domain modulation. Its overall process is shown in Figure 6.

Figure 6 illustrates the overall process of phase-guided structural reconstruction and spatial response. The structures in the figure are arranged from left to right according to the information flow. The left side represents the dual-modal input feature region, with the upper part containing the feature map extracted by the RGB branch and the lower part by the feature map extracted by the thermal infrared branch. These two feature maps converge at the feature fusion node to form a unified fused feature F . The fused feature then enters the frequency domain modeling stage, where it is mapped to a frequency domain representation through a Fourier transform module and decomposed into two parallel paths: an amplitude spectrum A(u,v) and a phase spectrum P(u,v) . The amplitude spectrum represents the overall frequency intensity with a smooth energy distribution, while the phase spectrum represents structural information with directional texture. The phase spectrum then enters the phase structure enhancement unit in a subsequent module. This unit enhances the structural components in the frequency domain through high-frequency response modulation, resulting in a more pronounced structural response in the high-frequency region after enhancement. The amplitude $\tilde{P}(u,v)$ spectrum maintains its original energy distribution and propagates along the parallel path. In the structural reconstruction stage, the enhanced phase spectrum and the original amplitude spectrum are recombined in the phase-guided reconstruction module to form reconstructed frequency domain features $F_{r}(u,v)$ . These features are then mapped back to the spatial domain through an inverse Fourier transform, yielding the enhanced structural features $F_{s}(x,y)$ . This feature map presents a clearer structural outline in the target boundary region. The spatial domain structure enhancement features and the original fused features F are jointly modulated in the phase modulation fusion module. The final fused features are formed through element-wise modulation and residual superposition $F_{f}$ , and are then used for salient target prediction. The overall process establishes a closed-loop structure between frequency domain structure enhancement and spatial domain feature modulation, enabling the boundary information of the target region to form a stable response in the spatial features, while suppressing interference from irrelevant structures in the background region.

### 2.5 Multi-scale supervision and optimization strategy

During training, PSRNet jointly optimizes salient-region localization, boundary integrity, and cross-modal structural consistency. Because a single output scale cannot fully constrain deep semantics and shallow boundary details, the decoder produces multi-scale saliency predictions, and each scale is supervised by pixel-wise, structural-gradient, and phase-alignment losses.

During the decoding phase, the network generates saliency map prediction results from features at different levels. Let the input image size be H×W. The decoder generates four saliency maps $S_{1},S_{2},S_{3},\text{and}\;S_{4}$ with spatial resolutions $\frac{H}{4}\times \frac{W}{4}$ , $\frac{H}{8}\times \frac{W}{8}$ , $\frac{H}{16}\times \frac{W}{16}$ and $\frac{H}{32}\times \frac{W}{32}$ , respectively. All predictions are upsampled to H×W by bilinear interpolation. Let $G\in R^{H\times W}$ denote the ground-truth saliency map with pixel values in [0,1] .

The prediction error of salient regions is constrained by pixel-level binary cross-entropy loss, as shown in Equation 17:


$$L_{\mathrm{bce}}(S_{i},G)=-\frac{1}{\mathrm{HW}}\sum_{x=1}^{H}\sum_{y=1}^{W}[\begin{array}{l} G(x,y)\log S_{i}(x,y)\\ +(1-G(x,y))\log(1-S_{i}(x,y)) \end{array}]$$ (17)

Equation 17, $S_{i}(x,y)$ denotes the saliency probability predicted at the *i*-th scale and pixel location (x,y) ; G(x,y) denotes the corresponding ground-truth label; and H and W denote image height and width, respectively. This loss function constrains the prediction of salient regions through pixel-wise error accumulation.

In salient target detection tasks, structural information is mainly reflected in the boundary gradient distribution. Therefore, a structure preservation constraint is introduced during the supervision process, and the structural difference between the predicted map and the true annotation is limited by the gradient consistency loss. Suppose that the two-dimensional gradient operator consists ∇ of horizontal gradient $\nabla_{x}$ and vertical gradient $\nabla_{y}$ , then the difference between the predicted structural gradient and the true structural gradient is shown in Equation 18:


$$L_{\mathrm{str}}(S_{i},G)=\frac{1}{\mathrm{HW}}\sum_{x=1}^{H}\sum_{y=1}^{W}|\nabla S_{i}(x,y)-\nabla G(x,y)|$$ (18)

In Equation 18, $\nabla S_{i}(x,y)$ represents the gradient magnitude of the predicted saliency map at the pixel location (x,y) , ∇G(x,y) while the other represents the gradient magnitude of the true saliency label at that location. This constraint measures the consistency of the target structure boundary through gradient difference, ensuring that the prediction results maintain a continuous structural response in edge regions.

Since the network introduces frequency-domain phase modeling, the same amplitude-weighted circular phase-distance formulation is applied at each supervised scale. Let Phi^R_l and Phi^T_l denote the RGB and thermal phase features at the l-th scale, and let M_l denote the number of spectral elements at that scale. This revised notation keeps Equation 19 consistent with Equation 11 and avoids using a direct absolute difference for circular phase variables.


$$L_{\mathrm{Pha}}^{\left(i\right)}=\frac{1}{M_{i}}\sum_{k}w_{i}(k)|\sin(\Phi_{i}^{R}(k)-\Phi_{i}^{T}(k))|.$$ (19)

In Equation 19 Let represent the i- k th element in the frequency domain feature vector k , represent N the total dimension of the frequency domain features, $\Phi_{i}^{R}(k)$ and $\Phi_{i}^{T}(k)$ represent the phase values of the RGB and thermal infrared features in that dimension, respectively. This constraint maintains consistency in the bimodal structure representation, ensuring that the phase structure information remains stably aligned during training.

Multi-scale supervision achieves the overall optimization goal by applying uniform constraints to all prediction levels. Let *Ns* denote the number of supervised decoder scales, then the total loss function is shown in Equation 20:


$$L_{\text{total}}=\sum_{i=1}^{M}(\begin{array}{l} \lambda_{1}\;L_{\mathrm{bce}}(S_{i},G)+\lambda_{2}\;L_{\mathrm{str}}(S_{i},G)\\ +\lambda_{3}\;L_{pha}\;(\Phi_{i}^{R},\Phi_{i}^{T}) \end{array})$$ (20)

In Equation 20, L_total denotes the overall optimization objective, n denotes the number of supervised decoder scales, and M_l denotes the total number of spectral elements used when averaging the phase loss at scale l. The weights lambda_b and lambda_p control the relative contributions of gradient-based boundary preservation and amplitude-weighted phase consistency, respectively.

During training, the backpropagation algorithm is used for $L_{\text{total}}$ minimization optimization, ensuring that salient region localization, structural boundary representation, and cross-modal structural consistency converge under a unified optimization objective. A multi-scale supervision mechanism continuously constrains structural representation at different semantic levels, enabling the network to form a stable structural propagation path between deep semantics and shallow details, thereby maintaining the structural integrity and boundary clarity of salient targets.

## 3 Experiment

### 3.1 Dataset and evaluation metrics

In the experimental section, the selected RGB-T salient object detection benchmark datasets were first systematically reviewed, and the number of images, scene categories, resolution range, and annotation methods of each dataset were statistically analyzed to clarify the data foundation for model evaluation. Based on the above statistics, Table 1 was compiled.

**Table 1:** Dataset composition.

Dataset	Images	Categories	Resolution range	Annotation type
VT821	821	21	240 × 320 to 720 × 1,280	Binary mask
VT1000	1,000	25	360 × 480 to 1,080 × 1920	Binary mask
VT2000	2,000	30	240 × 320 to 1,280 × 720	Binary mask
VT5000	5,000	45	240 × 320 to 1920 × 1,080	Binary mask
VT3000	3,000	38	360 × 480 to 1,024 × 768	Binary mask

This table lists the names of five publicly available datasets, the number of corresponding images, the number of scene categories, the upper and lower bounds of image resolution, and the pixel-level labeled categories. Each dataset covers sample sizes ranging from 821 to 5,000 pairs, scene categories ranging from 21 to 45, and resolutions ranging from a minimum of 240 × 320 to a maximum of 1,920 × 1,080. All datasets provide ground truth annotations in the form of binary masks to support subsequent model training and evaluation.

### 3.2 Implementation details

The experimental phase of this study focused on the hyperparameter settings, data preprocessing procedures, and hardware computing resource configuration for network training. Within a unified framework, key elements such as optimizer type, learning rate decay strategy, input scale normalization method, and loss function combination were standardized to ensure fairness and reproducibility during training for different comparison methods. For the RGB-T bimodal input characteristics, the data augmentation stage simultaneously processed visible light and thermal infrared images to maintain spatial correspondence, while batch size and gradient backpropagation mechanism were determined based on memory constraints. The training cycle, weight decay intensity, and convergence criteria were all initialized based on validation set feedback. Detailed configuration items are listed in Table 2.

**Table 2:** PSRNet training configuration details.

Parameter	Value	Parameter	Value
Optimizer	AdamW	Initial LR	1e-4
LR Schedule	Poly	Batch size	16
Epochs	200	Input size	352 × 352
Loss function	IoU + BCE	Weight decay	1e−4
Data augmentation	Random flip/crop/color jitter	Hardware	RTX 3080

This configuration table summarizes the core parameter settings and execution environment information relied upon during the network training phase. The optimizer uses AdamW to accommodate the coupled requirements of weight decay and adaptive learning rate adjustment; the learning rate smoothly decreases with iteration under a polynomial decay strategy. The number of input samples per batch is fixed at 16, and the input images are uniformly scaled to 352 pixel resolution using bilinear interpolation to balance memory usage and detail preservation. The loss function is composed of an intersection-over-union constraint and a binary cross-entropy, and the weight decay coefficient is applied to the non-normalized layer parameters to suppress overfitting. The data augmentation pipeline includes three types of operations: random horizontal flipping, random cropping, and color space perturbation; the same geometric transformation is applied synchronously to the thermal infrared modality. The computing platform is equipped with an NVIDIA RTX 3080 graphics processor, supporting the end-to-end optimization process under the above settings.

### 3.3 Comparison methods and experimental setup

This section focuses on the selection of comparison methods and experimental configuration, emphasizing the standardized settings followed for fair performance comparisons with existing RGB-T RGB-T salient object detection models. The experiments include representative methods published in authoritative journals and conferences in recent years, covering different backbone network architectures and multimodal fusion mechanisms to construct a comprehensive and discriminative evaluation baseline. All methods involved in the comparison were retrained or inferred based on a unified data partitioning and training strategy, without introducing additional intervention factors such as test-time enhancements to eliminate the potential impact of implementation differences on quantitative indicators. Basic information and core configuration principles of each comparison method are summarized in Table 3 below.

**Table 3:** Overview of comparison methods and experimental setup.

Method	Venue	Backbone	Experimental setting principle
MIDD	CVPR 2021	VGG-16	Uniform retraining
APNet	IEEE TIP 2022	ResNet-50	Identical augmentation
CGMDRNet	AAAI 2023	Swin-T	Original configuration
ADFNet	ACM MM 2023	ResNet-50	Synchronized iterations
CAVER	CVPR 2024	PVT-v2	No TTA
Ours	–	ResNet-50	Standard augmentation
CSRNet	Recent RGB-T SOD	ResNet-50	Official/no TTA

The compared methods differ in terms of publication source, backbone, and experimental control conditions. Their design principles directly reflect the comparative experimental specifications implemented in this paper. MIDD uses VGG16 as the feature extraction basis and retrains uniformly under the current data partitioning. APNet and ADFNet both use the ResNet −50 skeleton and maintain consistency in data augmentation strategies and synchronization of training iterations, respectively. CGMDRNet uses the hyperparameter configuration disclosed in the original paper to preserve the original characteristics of the method. CAVER disables test-time augmentation during the testing phase to ensure the purity of the original model’s output evaluation. Our method only applies basic random flipping and cropping operations to conform to the conventional training paradigm. These settings ensure that each method outputs comparable quantitative results under the same evaluation framework.

## 4 Results analysis

### 4.1 Overall performance analysis

**Figure 7:**
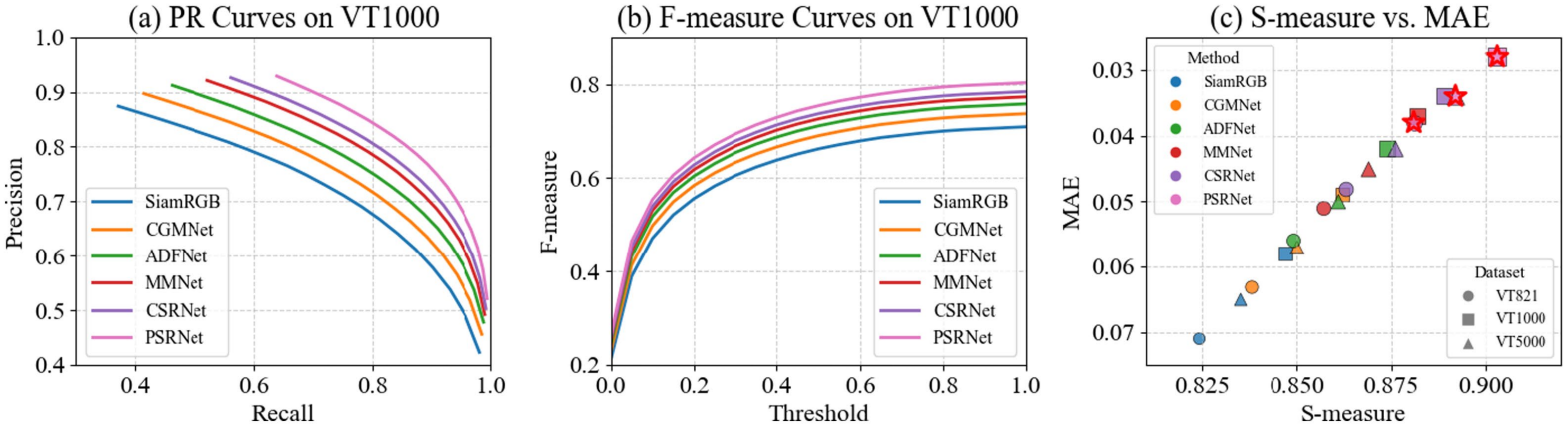
PSRNet overall performance multi-dimensional comparison analysis chart. **(a)** PR curve. **(b)** F-measure curve. **(c)** Scatter distribution of S-measure and MAE.

To comprehensively evaluate the structure preservation and cross-modal fusion capabilities of the proposed PSRNet in the RGB-T salient object detection task, this section conducts performance analysis from three dimensions: precision-recall tradeoff characteristics, comprehensive performance across multiple thresholds, and distribution of quantitative indicators across multiple datasets. Experiments were conducted on three benchmark datasets — VT821, VT1000, and VT5000— to uniformly test PSRNet and five representative comparative methods. The precision and recall sequences, F-measure dynamic curves, and three core indicators—S-measure, mean absolute error, and maximum F-measure—were recorded for each model under different thresholds. The results are shown in Figure 7.

Figure 7 presents the comprehensive performance evaluation. Figure 7a shows the precision-recall curves on VT1000, where PSRNet maintains a higher precision across the recall range of 0.639–0.994 and reaches a precision of 0.703 when recall is 0.940. The second-best CSRNet records 0.661 at the same recall point. Figure 7b reports F-measure values in ascending threshold order. PSRNet obtains 0.754, 0.779, and 0.794 at thresholds 0.50, 0.65, and 0.80, respectively, which are consistently higher than the compared methods. Figure 7c shows that PSRNet achieves an S-measure of 0.903 and an MAE of 0.028 on VT1000; on VT821 and VT5000, the corresponding S-measures are 0.881 and 0.892, with MAEs of 0.038 and 0.034. These results indicate stable improvements in structural similarity and error reduction without claiming statistical significance beyond the reported metrics.

### 4.2 Structural retention capability analysis

**Figure 8:**
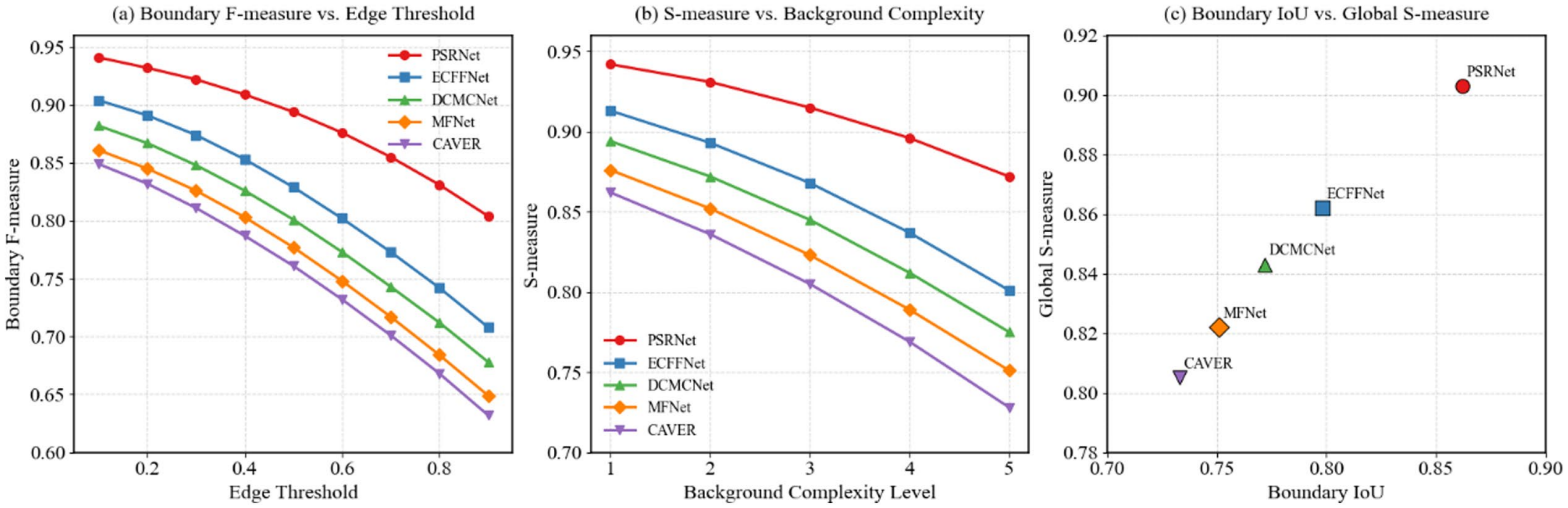
Structural retention capability analysis. **(a)** Boundary F-measure curves under different edge thresholds. **(b)** S-measure variation curves under different background complexity levels. **(c)** Scatter distribution of boundary IoU and global S-measure.

To evaluate PSRNet’s performance in terms of salient target boundary sharpness and structural integrity, this study designed three comparative analyses: the trend of boundary accuracy with edge threshold, the decay law of structural similarity index under different background complexities, and the joint distribution relationship between boundary intersection-over-union ratio and global structural measure. PSRNet and four mainstream comparative methods (ECFFNet, DCMCNet, MFNet, and CAVER) were systematically tested on RGB-T salient target detection benchmarks such as VT821 and VT1000. The boundary F-measure, S-measure under different background complexity levels, and corresponding values of boundary IoU and global S-measure were recorded for each method, resulting in the results shown in Figure 8.

Figure 8 compares structural preservation across edge thresholds, background complexity levels, and the joint distribution of boundary IoU and global S-measure. In Figure 8a, PSRNet records higher boundary F-measure values over the full threshold range, decreasing from 0.941 at threshold 0.1 to 0.804 at threshold 0.9. In Figure 8b, its S-measure decreases from 0.942 to 0.872 as background complexity increases from level 1 to level 5, corresponding to a smaller degradation than the compared methods. In Figure 8c, PSRNet obtains a boundary IoU of 0.862 and a global S-measure of 0.903, indicating coordinated improvement in boundary recovery and region-level structure preservation.

### 4.3 Analysis of cross-modal fusion effect

To evaluate the structural alignment quality and information complementarity of PSRNet in cross-modal fusion, experiments were conducted to compare the structural similarity distribution, phase consistency error, and boundary refinement dynamics during training under different fusion strategies. The contributions of phase constraints and reconstruction mechanisms to fusion performance were analyzed using ablation techniques. Based on these analyses, the comprehensive results, comprising three subgraphs, are shown in Figure 3.

Figure 3a compares four fusion strategies: RGB-only, T-only, simple fusion, and PSRNet. PSRNet reaches a mean SSIM of 0.89 with a standard deviation of 0.015, compared with 0.80 for simple fusion and 0.73/0.70 for RGB-only and T-only settings. Figure 3b shows that the peak phase consistency error decreases from approximately 0.45 without phase constraints to 0.22 with PSRNet, suggesting reduced cross-modal structural offset. Figure 3c shows smoother boundary-F1 convergence, with PSRNet reaching 0.920 after 100 epochs, compared with 0.757 for the baseline without reconstruction. These observations provide empirical support that latent phase modelling contributes to structural alignment in deep features, even though it is not treated as exact raw-image phase reconstruction.

To avoid over-claiming, the visualization and ablation results are interpreted as empirical evidence for deep-feature structural guidance, not as proof that raw-image Fourier phase is completely preserved after nonlinear transformations. This distinction is important for multimodal RGB-T perception, where the phase branch serves as an auxiliary alignment signal rather than a replacement for spatial semantic learning.

### 4.4 Module validity analysis

To verify the contribution of each module, this section reports a module-validity analysis for PSRNet. The analysis focuses on three ablated variants: w/o FD, w/o PC, and w/o SR. The revised analysis focuses on three ablated variants: removing the frequency-domain decoupling module (w/o FD), removing the phase consistency constraint (w/o PC), and removing the phase structure reconstruction module (w/o SR).

Figure 4a summarizes the multi-metric ablation comparison. The full PSRNet obtains 0.972, 0.903, 0.921, 0.935, and 0.865 for 1-MAE, S-measure, F-measure, E-measure, and IoU, respectively. Removing the phase structure reconstruction module reduces the F-measure to 0.880 and IoU to 0.815, indicating that bounded high-frequency structural reconstruction is central to boundary recovery. Removing frequency-domain decoupling yields an F-measure of 0.902, while removing phase consistency yields 0.890, showing that both spectral separation and cross-modal phase alignment contribute to the final performance. Figures 4b,c further confirm that the full model maintains a stronger precision-recall trade-off and a higher F-measure peak than the ablated variants (Table 4).

**Table 4:** Reorganized ablation summary based on the quantitative values reported in Figure 4 and Section 4.4.

Variant	Removed/modified component	1-MAE	S-measure	F-measure	E-measure	IoU
Full PSRNet	None	0.972	0.903	0.921	0.935	0.865
w/o FD	Frequency-domain decoupling	–	–	0.902	–	–
w/o PC	Phase consistency constraint	–	–	0.890	–	–
w/o SR	Phase structure reconstruction	–	–	0.880	–	0.815

### 4.5 Robustness analysis

**Figure 9:**
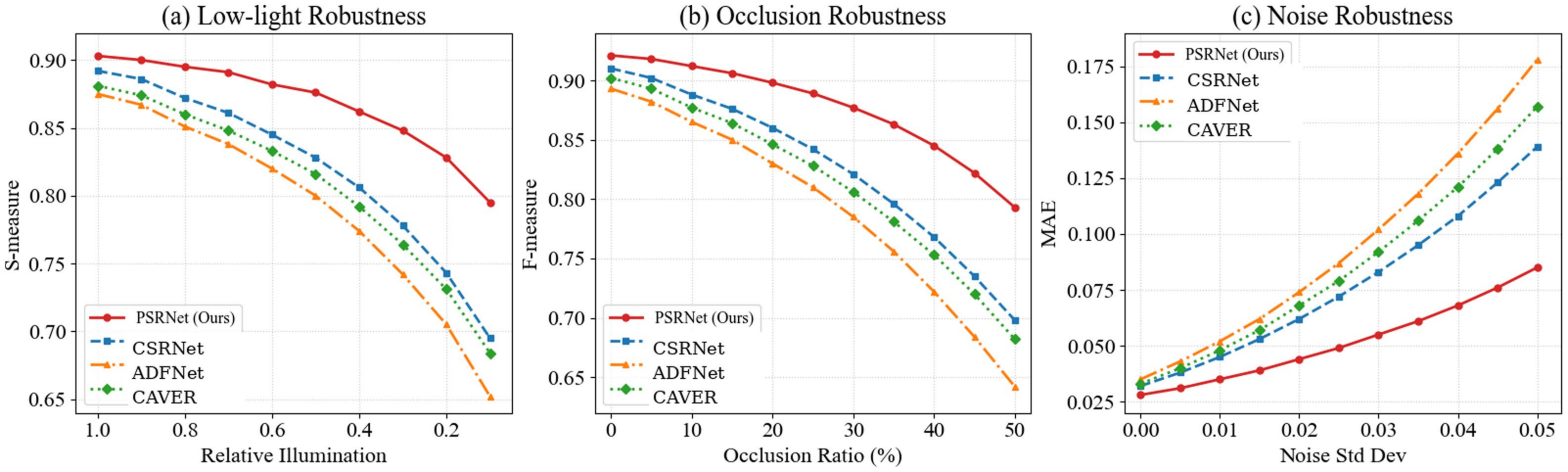
Robustness analysis results. **(a)** Changes in S-measure under low light conditions. **(b)** F-measure change under occlusion ratio. **(c)** MAE variation under noise intensity.

To evaluate the stability of the model under different degradation conditions, this section conducts a robustness analysis of PSRNet and three comparative methods from three perspectives: low illumination, occlusion, and noise interference. Experiments were conducted by varying the relative illuminance, occlusion ratio, and Gaussian noise standard deviation, recording the continuous changes in the S-measure, F-measure, and MAE indices for each method. The results are shown in Figure 9.

Figure 9a shows that when the relative illumination decreases from 1.00 to 0.10, the S-measure of PSRNet decreases from 0.903 to 0.795, while CSRNet, ADFNet, and CAVER decrease to 0.695, 0.652, and 0.684, respectively. Figure 9b shows that when the occlusion ratio increases to 50%, PSRNet records an F-measure of 0.793, which is 0.151 higher than ADFNet under the same condition. Figure 9c shows that when the Gaussian noise standard deviation reaches 0.05, PSRNet obtains an MAE of 0.085, compared with 0.157 for CAVER.

## 5 Conclusion and future work

This paper proposed PSRNet, a phase-guided frequency-domain structure reconstruction network for RGB-T salient object detection. The method maps bimodal deep features into the frequency domain, decouples amplitude and latent phase components, aligns reliable cross-modal structural cues through an amplitude-weighted phase consistency constraint, and reconstructs boundary-sensitive high-frequency details through a bounded Gaussian high-pass-guided operator. The reconstructed structure is then fed back into spatial features by adaptive phase-modulated fusion and optimized under multi-scale supervision. Experimental results show consistent gains in structure preservation and boundary recovery. On VT1000, PSRNet achieves an S-measure of 0.903, an MAE of 0.028, and an F-measure of 0.794 at threshold 0.80; the boundary IoU reaches 0.862 with a global S-measure of 0.903. Cross-modal fusion analysis further shows a mean SSIM of 0.89 and a reduction in peak phase consistency error from 0.45 to 0.22. These findings indicate that bounded latent-phase structural modelling can improve RGB-T SOD under complex backgrounds, low illumination, occlusion, and noise. Future work will further examine statistical significance across repeated runs, adaptive spectral filters, and broader multimodal perception tasks.

## Data Availability

The original contributions presented in the study are included in the article; further inquiries can be directed to the corresponding author.
